# Normal bone mineral density and bone microarchitecture in adult males with high and low risk of exercise addiction

**DOI:** 10.3389/fspor.2022.1021442

**Published:** 2022-11-14

**Authors:** Stinus Gadegaard Hansen, Mia Beck Lichtenstein, Karen Krogh Johansen, Rene Klinkby Støving

**Affiliations:** ^1^Department of Diabetes and Endocrinology, University Hospital Southern Denmark, Esbjerg, Denmark; ^2^Department of Regional Health Research, University of Southern Denmark, Odense, Denmark; ^3^Center for Digital Psychiatry, Mental Health Services in the Region of Southern Denmark, Odense, Denmark; ^4^Center for Eating Disorders, Odense University Hospital, Research Unit for Medical Endocrinology, Odense University Hospital, Mental Health Services in the Region of Southern Denmark, Odense, Denmark; ^5^Department of Clinical Research, University of Southern Denmark, Odense, Denmark

**Keywords:** exercise addiction, bone mineral density (BMD), bone microarchitecture, testosterone, leptin

## Abstract

Exercise addiction describes a pattern of excessive and obsessive exercise and is associated with hypoleptinemia and low testosterone that may have adverse skeletal effects. We used a validated questionnaire to identify males with high and low risk of exercise addiction. In a cross-sectional design, males (aged 21–49 years) with high (*n* = 20, exercise addictive) and low risk (*n* = 20, exercise controls) of exercise addiction had examinations of bone mass, bone microarchitecture, and estimated bone strength performed using dual-energy x-ray absorptiometry of the hip and spine and high-resolution peripheral quantitative computed tomography of the distal radius and tibia. Findings were compared between the groups and to a population-based sample of healthy men aged 20–80 years (*n* = 236). We found similar hip and spine bone mineral density in exercise addictive and controls. Cortical and trabecular bone microarchitecture and estimated bone strength in radius and tibia did not differ significantly between the groups. Multiple regression analyses adjusting for age, body weight, free testosterone, and hours of weekly training did not alter findings. Also, bone indices from both groups were within 95% prediction bands derived from the population-based sample for the vast majority of indices. Neither group had no associations between circulating leptin or free testosterone and bone outcomes. In conclusion, in a study on younger males, we found no associations between high risk of exercise addiction and various indices of bone mass and bone quality indicative of altered skeletal health.

## Introduction

Exercise and sports activities have numerous positive effects on physical and mental health. However, a minority develops a pattern of excessive and obsessive exercise that may have detrimental effects on health (1). The term exercise addiction has been proposed that in accordance with other behavioral addictions (such as gaming addiction and internet addiction), includes the symptoms of (1) salience where exercise becomes the most important thing in life, (2) tolerance where increasing amounts of exercise are required to achieve an effect, (3) mood modification where exercise is used as a coping strategy to regulate emotions, (4) conflicts with others about exercise patterns, (5) withdrawal symptoms when exercise cannot be performed, and (6) loss of control including the inability to limit time given to exercise (2).

Overtraining syndrome refers to excessive exercise without adequate recovery with a risk of injuries to bones, muscles, ligaments, or tendons (3). While such injuries are readily recognized, excessive exercise may have other, more subtle effects on physical health outplayed through the endocrine system.

In a cohort of adult males (21–49 years) with a high risk of exercise addiction, we have previously found that exercise addiction was associated with lower levels of free testosterone compared to males with a low risk of exercise addiction indicative of altered signaling in the hypothalamic-pituitary-gonadal (HPG) axis (4). In addition, circulating leptin was lower in exercise addictive even after adjusting for body fat percentage. In females exerting large amounts of exercise, alteration in the HPG axis has long been recognized as part of the *female athlete triad* that describes the phenomena of low energy availability (with or without disordered eating), menstrual dysfunction, and decreased bone mineral density (BMD) (5). Energy availability is defined as the dietary energy available to sustain physiological functions after subtracting the energetic cost of exercise (6). In the pathophysiological model of the female athlete triad, low energy availability was considered essential for the observed changes and could be estimated as the difference between energy intake and exercise energy expenditure relative to lean body mass. This also includes a state of relative leptin deficiency and treatment with recombinant leptin in females with hypothalamic amenorrhea significantly improved luteinizing hormone levels and pulse patterns and restored ovulation (7). Whether a phenomenon analog to the female athlete triad occurs in males, who exercise excessively is largely unstudied (8).

Testosterone is the main androgen in males and its physiological effects are important for skeletal development, bone accrual and maintenance (9). Males with acquired hypogonadism have increased bone turnover and bone resorption that may lead to severe bone loss, osteoporosis and increased risk of bone fractures (10).

Bone mass has long been recognized as a major determinant of bone strength and the measurement of BMD using dual-energy x-ray absorptiometry (DEXA) is widely used as a surrogate for bone mass to diagnose osteoporosis and assess fracture risk (11). In recent years, and parallel with the development of refined bone image diagnostics, it has become increasingly evident that bone characteristics besides bone mass, such as bone geometry and microarchitecture are also important determinants of bone strength (12). Now, advanced computed tomography techniques of the peripheral skeleton (such as high-resolution peripheral computed tomography, HR-pQCT) allow characterization of the bone microarchitecture in the outer (cortical) and inner (trabecular) compartments of the bone without the use of invasive techniques such as bone biopsy (13). Studies have documented a large impact on bone microarchitecture in various diseases (14, 15), changes with age (16) and between genders (17). In addition, such microarchitecture changes may not be reflected in assessments of bone mass and are therefore undetectable using DEXA. However, changes in microarchitecture may severely influence the biomechanical properties of bone and the resilience to fractures (18).

In this study, we aimed to explore bone health including bone mass and microarchitecture in adult males with a high risk of exercise addiction and biochemical indices suggestive of altered HPG axis regulation, in comparison to healthy males with a low risk of exercise addiction.

## Subjects and methods

### Participants

This cross-sectional study was performed at Odense University Hospital, Denmark after ethical approval from the Regional Health Ethics Committee of Southern Denmark. All participants provided informed consent.

Participants were recruited from a survey study advertising for male participants from fitness clubs in the city of Odense, from sports science and medicine faculties at the University of Southern Denmark, from the Funen Police Station, and from Internet advertisements. The inclusion criteria were regular exercise patterns. We included 20 males with a high risk of exercise addiction and 20 males with a low risk that served as controls. Males with a high risk of exercise addiction exercised on average 12 hours/week (range 6–18 h) and exercise controls exercised 5 hours/week (range 2–10 hours).

### Exercise addiction inventory

The Danish version of the Exercise Addiction Inventory 28 (EAI) was used to measure symptoms of exercise addiction. It consists of six items that are rated on a 5-point Likert scale from strongly disagree to strongly agree. Based on the classification of Terry et al. from 2004 (19) a total score of 24–30 indicates a high risk of exercise addiction. The EAI has been validated across several languages, including Danish, and has shown good psychometric properties (20).

### Anthropometrics

Body weight was measured using a Seca Model 708 scale (Seca, Hamburg, Germany) with participants dressed in indoor clothes and without shoes while height was measured using a wall-mounted stadiometer (Holtain Ltd, Crymych, United Kingdom).

### Biochemistry

Blood samples were collected between 8 and 9 AM after an overnight fast. As detailed earlier (4), we measured plasma testosterone using mass spectrometry (LS-MS System, Thermo Fisher Scientific Inc., Franklin, MA, USA) and sex-hormone binding globulin (SHBG) using an immunoassay (Auto-DELFIA, PerkinElmer, Waltham, MA, USA). Free testosterone was estimated as plasma testosterone/SHBG. Leptin was measured using an in-house immunofluorometric assay (21).

### Dual-energy X-ray absorptiometry (DEXA)

We measured areal bone mineral density (BMD) at the non-dominant hip (total hip region) and lumbar spine (L1–L4) using DEXA (Hologic, Waltham, MA, USA). Z-scores were calculated using reference values from males of similar age as participants as recommended by the International Society of Clinical Densitometry where z-scores < −2 were regarded as low bone mass for age. The CV in our unit for measurement of both the hip and spine is 1.5%.

### High-resolution peripheral quantitative computed tomography (HR-PQCT)

Bone geometry, volumetric bone mineral density (vBMD), microarchitecture and estimated bone strength at the distal radius and tibia were assessed using HR-pQCT (Xtreme CT I, Scanco Medical AG, Brütisellen, Switzerland). Method validation, image acquisition and analyses have previously been described in detail (22). In brief, we applied the standard protocol for *in vivo* scanning providing a 9.02 mm 3D representation of the distal radius and tibia in the axial direction. An offset from the bone endplate to the beginning of the scan region of 9.5 mm in radius and 22.5 mm in tibia was used. Each image contains 110 slices with an isotropic voxel size of 0.082 mm. The operator immediately evaluated the most distal slice after acquisition for motion artifacts, and at each skeletal site up to two repeat scans were performed if necessary. After image reconstruction image quality was graded by one of the authors (SH) using a 5-step scale as suggested by the manufacturer (1 = best; no signs of motion artifacts, 5 = worst; severe motion artifacts) and images graded 4 or 5 were excluded from the analyses (23). Standard evaluation software (Scanco Medical Software v6.0) was used for the calculation of bone geometry (total, trabecular and cortical area), compartmental volumetric BMD (total, trabecular and cortical vBMD) and trabecular microarchitecture (bone volume per tissue volume (BV/TV), trabecular number, thickness and separation). We used extended cortical evaluation software to obtain indices of cortical thickness and cortical porosity (24). Last, we estimated the bone strength of the distal radius and tibia using a finite element solver (Finite Element Analysis Software v1.15, Scanco Medical). In a virtual model of the bone, an axial compression test is simulated and the load required to fracture the bone is estimated (failure load) (25). Quality control was performed with daily and weekly scans of phantoms (QRM, Möhrendorf, Germany). The CVs were up to 0.8% for densities, 5.0% for indices of trabecular microarchitecture, 7.2% for cortical indices and 1.7% for estimated failure load (26).

### Statistical analyses

Mean and standard deviation was calculated when data were normally distributed and median and range when they were not. Bone variables in exercise addictive and controls were compared using a *t*-test or Wilcoxon's rank sum test depending on whether data were normal or non-normally distributed, respectively. Since age was not similar between the groups and body weight tended to be lower in exercise controls, and since both age and weight are major determinants of BMD and bone microarchitecture, multiple regression analyses were performed with age and weight as covariates. In addition, to assess the effect of testosterone and hours of weekly training these variables were also included in the model. Model assumptions were checked using histograms and quantile-quantile plots of residuals. Transformation of the dependent variable was performed where necessary (log or square root, as appropriate). Spearman correlation analyses were performed to assess associations between BMD, HR-pQCT indices, leptin and free testosterone in exercise addictive and exercise controls. In addition, a graphic presentation of findings in exercise addictive and exercise controls was shown in comparison to a population-based sample of healthy men aged 20–80 years (*n* = 236) that previously participated in a study on age- and sex-related differences in bone microarchitecture (17) with 95% prediction bands. The study sample size was determined based on assumptions regarding circulating leptin (4) and the bone outcomes presented here were secondary outcomes and planned for explorative purposes rather than for hypothesis testing. We used Stata Statistical Software release 16 for statistical analyses and graphics (StataCorp LP, College Station, Texas, USA). A *p*-value below 0.05 was considered statistically significant.

## Results

Participants with exercise addiction were significantly younger (31 ± 8 years) than exercise controls (38 ± 7 years, *p* = 0.006) and tended to have lower body weight and body mass index (79 ± 8 kg and 24 ± 2.1 kg/m^2^) than exercise controls (86 ± 14 kg and 25.5 ± 3.1 kg/m^2^) although these differences were not statistically significant (*p* = 0.06 and 0.09, respectively) (Table 1).

**Table 1:** General and biochemical characteristics of exercise addictive and exercise controls.

	**Exercise addictive**	**Exercise controls**	***p*-value**
*n*	20	20	
Age, years	31 ± 8	38 ± 7	0.006
Height, cm	182 ± 6	184 ± 6	0.31
Weight, kg	79 ± 8	86 ± 14	0.06
Body mass index, kg/m2	24.0 ± 2.1	25.5 ± 3.1	0.09
Exercise, h/week	11.8 ± 3.8	4.8 ± 2.8	0.001
Free testosterone	0.46 ± 0.15	0.56 (0.15)	0.038
Leptin, ug/l	1.12 ± 1.31	4.26 ± 2.85	0.001

### DEXA

One participant in each group had a Z-score below −2 at either the spine or the total hip. There were no differences in the lumbar spine or the total hip BMD between exercise addictive and exercise controls (Table 2).

**Table 2:** Bone mineral density by DEXA and bone microarchitecture by HR-pQCT in exercise addictive and exercise controls.

	**Exercise addictive**	**Exercise controls**	***p*-value**	**Adjusted *p*-value#**
**DEXA (** ***n*** **)**	20	20		
Lumbar spine, g/cm^2^	1.02 ± 0.10	1.05 ± 0.16	0.57	0.62
Lumbar spine z-score	0.6 ± 0.9	0.4 ± 1.4	0.56	0.61
Total hip, g/cm^2^	1.06 ± 0.14	1.05 ± 0.12	0.88	0.97
Total hip z-score	−0.3 ± 0.9	−0.2 ± 0.8	0.88	0.96
**HR-pQCT**				
RADIUS (*n*)	19	19		
Total area, mm^2^	385 ± 42	389 ± 75	0.83	0.39
Cortical area, mm^2^	77 ± 12	73 ± 17	0.44	0.15
Trabecular area, mm^2^	303 ± 37	310 ± 73	0.70	0.62
Total vBMD, mg HA/cm^3^	337 ± 37	342 ± 58	0.74	0.63
Cortical vBMD, mg HA/cm^3^	874 ± 19	871 ± 49	0.82	0.24
Trabecular vBMD, mg HA/cm^3^	191 ± 30	201 ± 30	0.31	0.96
Cortical thickness, mm	0.99 ± 0.11	0.95 ± 18	0.44	0.33
Cortical porosity, %	1.50 (0.83–3.13)	1.86 (1.04–3.71)	0.19	0.23
BV/TV, %	15.9 ± 2.4	16.8 ± 2.6	0.31	0.97
Trabecular number, 1/mm	2.00 ± 0.20	2.09 ± 0.25	0.27	0.65
Trabecular thickness, mm	0.080 (0.055–0.089)	0.079 (0.063–0.114)	0.82	0.79
Trabecular separation, mm	0.43 ± 0.06	0.41 ± 0.06	0.29	0.79
Failure load, *N*	6,050 ± 185	6,171 ± 304	0.74	0.49
TIBIA (*n*)	20	20		
Total area, mm^2^	921 ± 143	951 ± 154	0.53	0.47
Cortical area, mm^2^	174 ± 28	172 ± 29	0.79	0.88
Trabecular area, mm^2^	749 ± 132	780 ± 161	0.51	0.47
Total vBMD, mg HA/cm^3^	345 ± 32	344 ± 49	0.99	0.69
Cortical vBMD, mg HA/cm^3^	886 ± 28	885 ± 31	0.94	0.14
Trabecular vBMD, mg HA/cm^3^	222 ± 27	221 ± 28	0.71	0.60
Cortical thickness, mm	1.35 ± 0.17	1.38 ± 0.23	0.59	0.74
Cortical porosity, %	4.60 ± 1.66	5.11 ± 1.77	0.36	0.758
BV/TV, %	18.1 ± 2.22	18.4 ± 2.32	0.70	0.60
Trabecular number, 1/mm	2.17 ± 0.23	2.27 ± 0.22	0.16	0.23
Trabecular thickness, mm	0.084 ± 0.083	0.081 ± 0.075	0.26	0.54
Trabecular separation, mm	0.38 ± 0.05	0.36 ± 0.04	0.19	0.23
Failure load, *N*	15,667 ± 2,467	16,143 ± 2,037	0.51	0.86

#multiple linear regression adjusting for age, body weight, free testosterone and hours of weekly training.

### HR-pQCT

Two radius images were excluded from analysis due to poor image quality (one in each group) while all other images had adequate quality. In both radius and tibia, total bone area was similar between exercise addictive and controls as were the compartmental geometry with similar cortical and trabecular areas (Table 2). Likewise, total, cortical and trabecular vBMD did not differ significantly between groups at either site. In terms of cortical microarchitecture, there were no differences in cortical thickness and cortical porosity between groups in radius or tibia as was the case for trabecular microarchitecture where BV/TV, trabecular number, thickness, and separation were similar at both sites.

**Figure 1:**
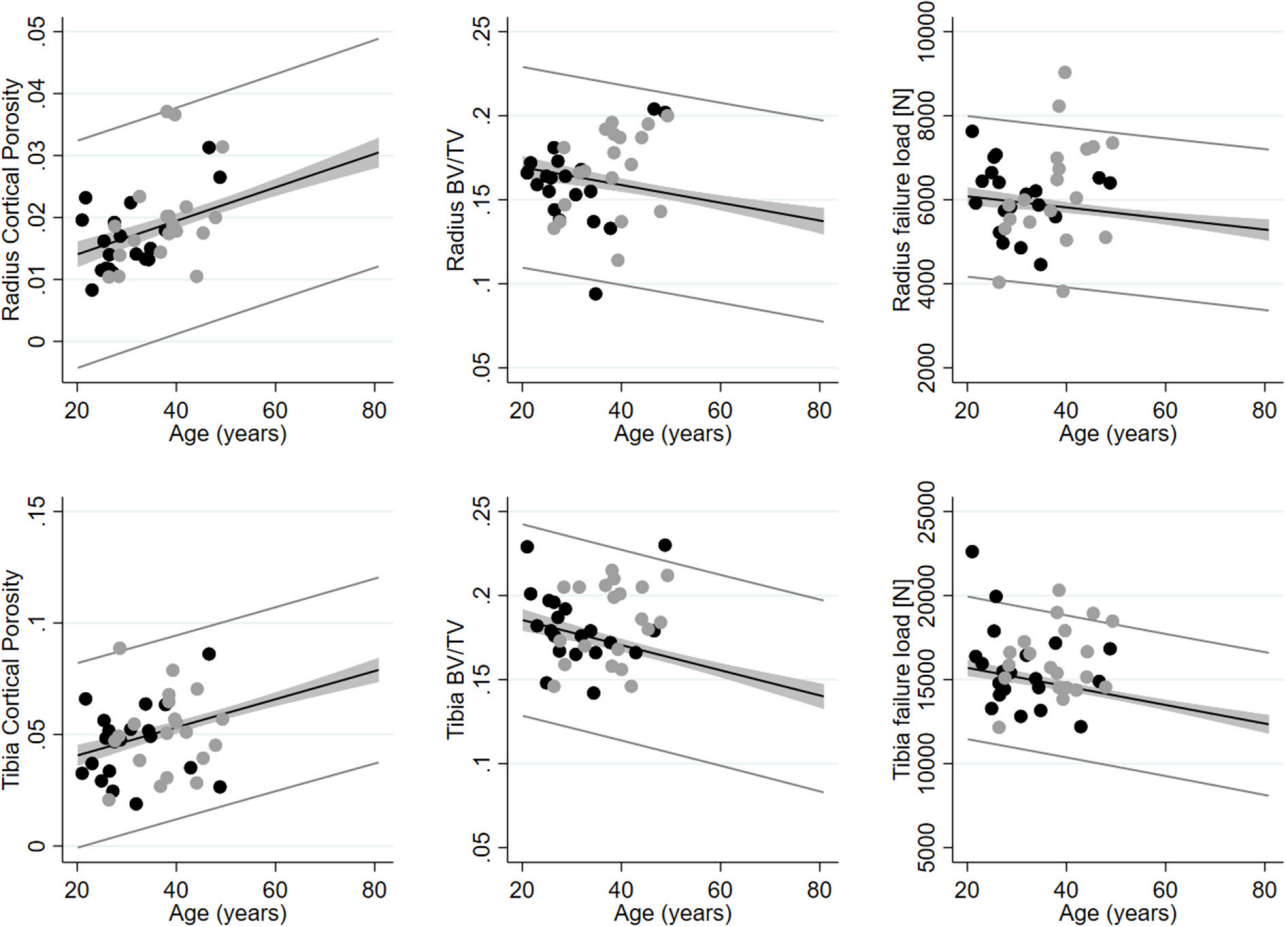
Cortical porosity, trabecular bone volume per tissue volume (BV/TV) and estimated bone failure load assessed using HR-pQCT in radius (upper row) and tibia (lower row) in exercise addictive (black dots) and exercise controls (gray dots). Shown with median and 95% upper and lower prediction bands using data from a population-based sample of healthy men aged 20–80 years (*n* = 236).

In graphical presentations of DEXA and HR-pQCT with median and 95% upper and lower prediction bands, derived from the population-based sample of healthy men, exercise addictive were within the 95% prediction bands for the vast majority of variables (Figure 1). In one case, radius trabecular vBMD and BV/TV were below the lower 95% prediction band while values were above the upper 95% prediction band for tibia BV/TV (*n* = 1) and tibia failure load (*n* = 2). Similarly, a few indices in exercise controls were outside the 95% prediction bands (Figure 1).

### Multiple linear regression analyses and spearman correlation analyses

In multiple regression analyses comparing bone outcomes between exercise addictive and exercise controls adjusting for age, body weight, free testosterone and hours of weekly training we did not find significant differences in either DEXA or HR-pQCT indices (Table 2). Likewise, there were no significant correlations between free testosterone and DEXA or HR-pQCT outcomes in either exercise addictive or exercise controls. Similarly, circulating leptin was not correlated with DEXA or HR-pQCT indices.

## Discussion

In this cross-sectional study, we assessed skeletal health with measurements of bone mass and microarchitecture using state-of-the-art bone imaging diagnostics in young men with a high risk of exercise addiction in comparison to peers with a low risk of exercise addiction. We did not find differences in BMD in the lumbar spine or hip nor cortical or trabecular microarchitecture or estimated bone strength in the distal radius and tibia between the groups. Similarly, no differences were found between groups in multiple regression analyses adjusting for effects of age, body weight, free testosterone and hours of weekly training. Thus, neither the exercise regimens performed in exercise addictive nor the observed hormonal changes in testosterone nor leptin between the groups appeared to influence skeletal health to any significant degree.

Bone is a dynamic tissue that through the closely regulated cellular process of bone remodeling, can adapt its shape, mass and internal distribution of bone to the mechanical demands applied to it (27). Accordingly, a large body of evidence documents numerous, positive effects of sporting activities on bone health (28). Running was the most popular exercise activity in both groups in our study while the remaining exercise activities did not differ essentially. Different exercise types have varying impacts on the adaptive mechanism in the bone tissue and ultimately bone strength (29). Weight-bearing exercises with gravitational bone loading as a result of impact with the ground (such as running or soccer) are generally superior to exercises without ground-reaction forces (such as cycling or swimming) for improving BMD, especially at the hip (30, 31). Based on the extensive training regimens in exercise addictive, one might expect these to have superior bone health compared to exercise controls. However, the majority in the addictive group were engaged in endurance sports such as running, cycling or swimming which generally have been found to have lower BMD than athletes participating in ball or power sports (32, 33). While endurance sports are normally beneficial for bone health, the osteogenic potential related to *elite* endurance sports is limited (32). In particular, low energy availability that is often observed in sports where leanness is pivotal for performance appears to exert detrimental effects on bone that outweigh the positive effects of training activities. To that end, a study on young men found that highly competitive master cyclists had significantly *reduced* BMD compared to both young adult elite cyclists and age-matched non-athletes (34). In our cohort, one in the exercise addictive group had a Z-score below −2 which was similar to that observed in exercise controls. Therefore, reductions in BMD to a level that would raise clinical concern were not overrepresented in the group of exercise addictive. In addition, the vast majority of indices of bone microarchitecture and estimated bone strength in exercise addictive were similar to those found in a sample of community-dwelling, healthy men of similar age. Thus, we did not find evidence that exercise addiction was associated with poorer bone health.

In older adults, complete withdrawal of testosterone causes a rapid increase in bone remodeling, a loss of bone mass and an increase in fracture risk (10). On the contrary, the effects of relative testosterone insufficiency on bone in younger men are less clear. Although exercise addictive in our study had lower levels of free testosterone compared to exercise controls, their free testosterone levels were within the reference interval, and none had a clinical diagnosis of hypogonadism. Interestingly, similar findings of low normal testosterone levels have been reported in male high-volume endurance athletes in several studies indicative of altered HPG axis regulation (8). However, evidence of an independent, negative effect of low normal testosterone on bone mass in males participating in elite sports is absent (8, 35) and nor did the present study demonstrate any negative effect. In accordance, most longitudinal cohort studies in eu-gonadal men with long follow-ups (6–18 years) did not find any association between testosterone level and fracture risk (9). Clinical experimental studies in young men using combinations of gonadotropin-releasing hormone (that blocks the HPG-axis and thereby gonadal testosterone secretion), aromatase inhibitors (that blocks the conversion of testosterone to estrogen in bone) and testosterone supplementation have shown that both estrogen and testosterone can independently block bone *resorption* and that testosterone plays an important role in the regulation of bone *formation* (36). A slight reduction in free testosterone within the normal range that appears evident in the exercise addictive in our study may, in theory, inflict minor negative changes in bone remodeling favoring bone resorption over bone formation leading to bone loss. However, we did not find associations between BMD or microarchitecture indices and free testosterone and the positive effects of mechanical stimuli related to exercise activities seem to have counterbalanced any negative effect related to relative testosterone insufficiency.

In our study, males with a high risk of exercise addiction had low circulating leptin compared to exercise controls. Low leptin has also been reported in other studies with exercising males with low energy availability (37) and young males in military training with low-calorie intake (38). Leptin was initially renowned for its role in energy homeostasis while it has become evident that it also plays an important role in neuroendocrine regulation and bone metabolism (39). Adipocytes secrete leptin and circulating levels generally signal the amount of body fat stores, although secretion drops acutely in response to low energy intake. Leptin regulates several pituitary hormonal axes through hypothalamic receptors and modulates the thyroid, adrenal, growth hormone and gonadal axis in humans all of which confer effects on bone metabolism and in general low circulating leptin leads to negative effects in bone (39). In correlation analyses, we did not find any associations between leptin and bone mineral density or microarchitecture status either in exercise addictive or across the two groups (data not shown). This implies that any independent effect of leptin was indeed minor and overshadowed by the numerous other determinants of bone health.

This was a small, cross-sectional study and several limitations should be mentioned. First, we had no information about the duration of exercise patterns in either the high-risk group or the low-risk group. Thus, we cannot assess the association between the duration of the exercise regimen and bone outcomes. Second, given the limited number of participants, sub-group analyses examining any specific impact of each sporting activity on bone outcomes were not possible. Third, information on energy intake was not obtained and therefore estimates of energy availability could not be calculated which precluded the exploration of low energy availability and bone outcomes. Fourth, the inability to show significant differences between the groups may relate to the study sample size. Although sound epidemiological data are lacking regarding the prevalence of exercise addiction this is a rare condition and recruiting a larger number of participants is challenging. With the number of participants included, we had the statistical power to detect a difference in i.e., lumbar spine Z-score of approximately 1 between the groups (alpha 0.05, beta 0.80) which in terms of fracture risk generally corresponds to a doubling in fracture risk (11). Thus, potential changes in BMD between groups below this threshold were undetected. To that end, we chose to include males only since this phenomenon is far less studied in males compared to females, and whether skeletal outcomes would be differently affected in females is unknown.

This study also has some strengths. We used a validated questionnaire to identify young men with a high risk of exercise addiction making the results generalizable to such populations. In addition, relevant control subjects were included that allowed comparisons with peers with low levels of exercise and with a population-based sample of men with a wide age span that permitted interpretation of results in relation to the general population.

In conclusion, we found no association between exercise addiction and bone health in a cohort of younger males. We found similar bone mass and microarchitecture in males with high and low risk of exercise addiction at a level that was comparable to that seen in a population-based sample from the general population. Although exercise addiction may lead to altered leptin and free testosterone levels, skeletal health is not affected to any significant degree. However, we recommend future research to investigate the long-term effect of excessive exercise on bone health.

## Data availability statement

The datasets presented in this article are not readily available because of legal restrictions. Requests to access the datasets should be directed to stinus.gadegaard.hansen@rsyd.dk.

## Ethics statement

This study was reviewed and approved by the Regional Health Ethics Committee of Southern Denmark. The participants provided their written informed consent to participate in this study.

## Author contributions

Conceptualization: ML and RS. Recruitment: ML, RS, and SH. Data management: SH and KJ. Statistical analysis and drafting of manuscript: SH. All authors revised the manuscript critically and approved the final version for publication.

## Conflict of interest

The authors declare that the research was conducted in the absence of any commercial or financial relationships that could be construed as a potential conflict of interest.

## Publisher's note

All claims expressed in this article are solely those of the authors and do not necessarily represent those of their affiliated organizations, or those of the publisher, the editors and the reviewers. Any product that may be evaluated in this article, or claim that may be made by its manufacturer, is not guaranteed or endorsed by the publisher.
